# Investigating the Quantification Capabilities of a Nanopore-Based Sequencing Platform for Food Safety Application via External Standards of Lambda DNA and Lambda Spiked Beef

**DOI:** 10.3390/foods13203304

**Published:** 2024-10-18

**Authors:** Sky Harper, Katrina L. Counihan, Siddhartha Kanrar, George C. Paoli, Shannon Tilman, Andrew G. Gehring

**Affiliations:** United States Department of Agriculture, Agricultural Research Service, Eastern Regional Research Center, Wyndmoor, PA 19038, USA; sky.harper@usda.gov (S.H.); katrina.counihan@usda.gov (K.L.C.); siddhartha.kanrar@usda.gov (S.K.); george.paoli@usda.gov (G.C.P.); shannon.tilman@usda.gov (S.T.)

**Keywords:** nanopore sequencing, foodborne pathogen quantification, real-time analysis, food testing

## Abstract

Six hundred million cases of disease and roughly 420,000 deaths occur globally each year due to foodborne pathogens. Current methods to screen and identify pathogens in swine, poultry, and cattle products include immuno-based techniques (e.g., immunoassay integrated biosensors), molecular methods (e.g., DNA hybridization and PCR assays), and traditional culturing. These methods are often used in tandem to screen, quantify, and characterize samples, prolonging real-time comprehensive analysis. Next-generation sequencing (NGS) is a relatively new technology that combines DNA-sequencing chemistry and bioinformatics to generate and analyze large amounts of short- or long-read DNA sequences and whole genomes. The goal of this project was to evaluate the quantitative capabilities of the real-time NGS Oxford Nanopore Technologies’ MinION sequencer through a shotgun-based sequencing approach. This investigation explored the correlation between known amounts of the analyte (lambda DNA as a pathogenic bacterial surrogate) with data output, in both the presence and absence of a background matrix (*Bos taurus* DNA). A positive linear correlation was observed between the concentration of analyte and the amount of data produced, number of bases sequenced, and number of reads generated in both the presence and absence of a background matrix. In the presence of bovine DNA, the sequenced data were successfully mapped to the NCBI lambda reference genome. Furthermore, the workflow from pre-extracted DNA to target identification took less than 3 h, demonstrating the potential of long-read sequencing in food safety as a rapid method for screening, identification, and quantification.

## 1. Introduction

The World Health Organization (WHO) estimates that each year, unsafe food causes 600 million cases of foodborne diseases and roughly 420,000 deaths worldwide [1]. Along with illnesses and deaths, the WHO estimates that foodborne diseases impose an economic burden, accounting for $110 billion annually in lost productivity and healthcare costs [2]. Another study [3] reported that the economic burden of foodborne illness may exceed $90 billion annually for the Unites States alone. Urbanization and consumer habits have increased the demand for a wider variety of food, resulting in an increasingly complex and longer global food chain, putting greater responsibility on food producers, handlers, and consumers to ensure food safety [2].

*Salmonella*, *Campylobacter* spp., pathogenic *Escherichia coli*, *Listeria monocytogenes*, *Yersinia enterocolitica*, and *Staphylococcus aureus* are among the most common foodborne pathogens, affecting millions of people annually. In some cases, these infections can result in severe and fatal outcomes [2,4,5,6,7,8]. According to the WHO, *Salmonella* is a key cause of diarrheal disease, with contamination linked to poultry and cattle products, along with dried foods and fruits and vegetables; with the severity of illness dependent on the specific serotype and strain [2,4]. While *Campylobacter* spp. may be the single most-common bacterial cause of diarrheal illness worldwide, they generally cause self-limiting gastroenteritis, with a higher risk of mortality in people experiencing secondary sequelae [5]. By contrast, the high mortality rate associated with infection by *L. monocytogenes* makes it a leading cause of death from foodborne illness, despite the relatively low incidence of illness [5]. Foodborne pathogenic *E. coli* comprise multiple diarrheagenic pathotypes, the deadliest of which are the Shiga toxin-producing strains that can cause hemorrhagic colitis and hemolytic uremic syndrome [6]. *Y. enterocolitica,* is a bacterium originating from meat animals, with pork and pork products being the primary source [7]. Propagation of *Y. enterocolitica* can occur at low temperatures, and it has been found to survive and reproduce in vacuum-packaged and refrigerated foods, including dairy, meat, poultry, fruits, vegetables, stewed and fermented products, and seafoods [7]. Similarly, *S. aureus* can be found in many food products, including raw retail meat, posing a risk of infection for consumers [8].

For decades, culture methods have been used as the primary technique for pathogen detection; however, the high costs of these lengthy, complex, and laborious procedures hinder their overall applicability [9,10]. The development and application of selective media has advantages in pathogen isolation and detection, though their use still requires significant incubation time, still leads to the growth of non-target flora, and makes absolute quantification impossible [9]. Immunological methods have reduced the time needed to detect pathogens; however, these methods are limited by the quality of antibodies and the requirement for high amounts of bacterial-based targets [9]. Furthermore, immunological methods are sensitive to in vitro test conditions and can lead to false-positive or false-negative results. In more recent years, molecular methods have been developed, such as PCR techniques, though they require highly trained labor, expensive instrumentation, and sample pre-treatments that continue to make field application difficult [9,10]. These various techniques can be effective in tandem and are often used for screening and identification, though quantification continues to remain an area of exploration.

Within the past 20 years, substantial developments have been made in nucleic acid sequencing, from first- and second-generation sequencing technologies to third-generation sequencing [11,12]. In 2014, Oxford Nanopore Technologies (ONT) released the MinION device, which is about the size of a thumb drive and capable of plugging directly into a standard USB port on a computer [13]. The device sequences RNA or DNA by detecting changes in electrical current as the nucleic acid passes through a protein nanopore on a flow cell, generating reads that the MinKNOW software can assemble for real-time analysis and sample identification [12,13]. Though the ONT MinION has a lower data output compared to other NGS technologies (e.g., Illumina or PacBio SMRT sequencing technologies), the significantly lower instrument cost, the smaller instrument size, portability, lower energy requirements, simplified library preparation, the ability to provide real-time sequence information with adaptive sampling, and potentially faster run times provide numerous advantages. All of these advantages, particularly instrument portability, raise the potential for rapid, on-site pathogen detection, particularly for samples for which culture enrichment could be avoided. Recent studies, in silico and in vitro, have investigated the application of the MinION for detection and characterization of pathogens such as *E. coli* O157:H7 and *L. monocytogenes* and concluded that sequencing provided multiple advantages over other methods [13,14,15,16,17,18,19]. Additionally, the small, portable ONT sequencers allow DNA or RNA sequencing and analysis to be conducted outside of traditional laboratories, and the cost is generally lower than second-generation sequencing, overcoming barriers that previous screening methods encountered [13]. The instrumental capability is revolutionary in the identification of virulence factors, antibiotic resistance genes, and other critical genomic attributes [13,20,21]. This aids in a deeper understanding of strain variations and contributes to targeted interventions. While current applications highlight the efficacy of nucleic acid sequencing in identification and characterization, traditional shotgun-based sequencing approaches have not been viewed as a methodology for quantification. The goal of this work was to evaluate the potential of the ONT MinION device for quantitative detection and identification of foodborne pathogens via a metagenomic shotgun-based sequencing approach using a model system employing bacteriophage lambda DNA as a pathogen surrogate in the presence and absence of bovine DNA.

## 2. Materials and Methods

### 2.1. Materials

The following materials were employed in this investigation: Nuclease Free Water and 200 Proof Molecular Grade Ethanol (GSA Advantage!, Washington, DC, USA), DNeasy Blood & Tissue Kit, (ID# 69504, Qiagen, Germantown, MD, USA), Control Expansion Kit (Part #: EXP-CTL001, Oxford Nanopore Technologies [ONT], Oxford, UK), Ligation Sequencing Kit v14 (SQK-LSK114, ONT), Flow Cells R10.4.1 (Part #: FLO-MIN114, Oxford Nanopore Technologies [ONT], Oxford, UK), and NEBNext Companion Module for Oxford Nanopore Technologies Ligation Sequencing (Part #: E71805, New England Biolabs Inc., Ipswich, MA, USA).

### 2.2. Bovine DNA Extraction from Ground Beef

*Bos taurus* DNA was extracted from a 5 g sample of irradiated ground beef from a local retailer. The sample was thawed and suspended in 500 µL of nuclease free water, homogenized using a vortex mixer, and processed using a Qiagen DNeasy Blood & Tissue Kit according to the manufacturer’s protocol. DNA concentration and quality measurements were taken with a DeNovix DS-11 FX+ spectrophotometer (DeNovix Inc., Wilmington, DE, USA). The resulting *Bos taurus* DNA sample had a concentration of 57.93 ng/µL and absorbance purity ratios ($\frac{260\;nm}{230\;nm}\;and\;\frac{260\;nm}{280\;nm})\;$ of 1.955 and 1.860, respectively. This sample was used as the source of background bovine DNA.

The composition of the bovine DNA sample was evaluated by sequencing 1000 ng of the bovine DNA in triplicate. The resulting 176,616 sequences were subject to a BLAST search, revealing only *Bos taurus* DNA.

### 2.3. MinION Sequencing

Libraries of purified lambda phage DNA from the ONT Control Expansion Kit were prepared using the Ligation Sequencing Kit V14 according to the manufacturer’s instructions [20]. Though the ONT-recommended input DNA for the Ligation Sequencing Kit V14 was 1 µg, variable amounts of lambda DNA were used for library construction (details below). Libraries of 1 µg of extracted *Bos taurus* DNA spiked with lambda phage DNA were also prepared with the Ligation Sequencing Kit v14 using the amounts indicated below [20]. Library preparation involved DNA end repair and dA-tailing using NEBNext End Repair/dA-tailing before sequencing adaptors were ligated to the prepared ends [22]. This kit was selected due to its potential ability to combine upstream processes such as target enrichment by capture, size selection, and multiplexing up to 96 samples with additional barcoding kits; however, for this investigation, these functions were not explored [22].

The initial experimental samples consisted of 80 ng, 200 ng, 600 ng, 1000 ng, and 1250 ng of lambda DNA generated from a lambda stock solution having a concentration of 50 ng/µL. Similarly, for the mixed DNA samples, the libraries consisted of a standard 1000 ng (1 µg) *Bos taurus* DNA input, spiked with 0 ng, 80 ng, 200 ng, 600 ng, 1000 ng, and 1250 ng of lambda DNA. All samples were prepared and run in triplicate. The amount of bovine DNA used (1 µg) is the amount of input DNA recommended by ONT. The amounts of lambda DNA used for analysis were selected to cover a greater than ~15-fold range but did not greatly exceed the recommended DNA input. This included amounts of lambda DNA less than 10% and greater than 50% of the total DNA in the mixed lambda/bovine samples.

Prior to sequencing, a flow cell check was performed to ensure that there were a sufficient number of pores available for sequencing. The MinION Mk1C (ONT) was used with R10.4.1 flow cells (ONT). For all runs, the default ONT MinION parameters were utilized, with the only changes being a minimum read length set to 20 bp, a minimum Q score of 8, and a 1 h run time. A one-hour sequencing time was used to expedite the entire screening and quantification pipeline for real-world consideration. The sequencing run time for all libraries was set for 1 h to reduce analysis time and improve the time-to-result.

For our investigations, the amount of bovine DNA employed aligns with that of the manufacturer’s recommendations for the MinION and thus was set at 1 µg, with varying amounts of lambda DNA. Thus, the input DNA varied from 80 ng to 2250 ng of total DNA, 1080 ng to 2250 ng for experiments including both lambda and bovine DNA, and 80 ng to 1250 ng for experiments with only lambda DNA.

### 2.4. DNA Analysis

Sequencing data for all samples were base-called in real-time via the MinKNOW software (ONT, version 23.04.5) on the MinION Mk1C using the “fast basecalling” model. The FastQ files were imported into Geneious Prime software (version 2023) and aligned to an *Enterobacteria* phage lambda complete genome (NCBI Accession #NC001416.1) using Minimap2 (version 2.24). In addition, the lambdap22 sequence from NC_001416.1 (NCBI Gene ID #2703502) was searched against the alignment to determine the number of reads that mapped to this target. The mean, standard deviation, and standard error of triplicate runs for each sample was determined for sequencing run general parameters and gene detection.

### 2.5. Statistical Methods

Several statistical tests were used to analyze the data derived from the ONT MinION. Spearman’s correlation coefficient was used to determine tonicity of the calibration curves to find an overall trend within the data [23]. A Grubbs test was then used to remove outliers from the data set, and one-way ANOVA tests were used to determine if data within a set showed significant difference. Pearson’s correlation coefficient was then used for relevant sets of data to determine the best fit line. The generated line was found through the method of least squares.

## 3. Results and Discussion

### 3.1. Sequencing Run Outputs for Lambda DNA Standard Curve

The first set of experiments followed the external standards calibration method, where a series of known sample concentrations is created, and the outputs are recorded. This series was then used to construct a calibration curve [24]. This is a simple method to determine if there is a relationship between MinION DNA input and data output, specifically, for lambda phage DNA in the absence of any background matrix. In this setup, only one known variable was changed, the amount of DNA, while a set time of 1 h was standard in all experiments.

The filtered general run outputs for this experiment are shown in Table 1. Standard deviations are listed for each output and were calculated after outlier removal based on the Dixon’s Q-test [25].

The results revealed a positive correlation with increasing DNA concentration for several parameters (Table 1). The data for each output was fitted to a line of best fit following the linear equation:

(1) $$y=\beta_{0}{+\beta }_{1}x$$

where *y* is the output signal/parameter, $\beta_{0}$ and ${\;\beta }_{1}$ are the y-intercept and slope of the line, respectively, and *x* is the input DNA concentration.

A significant positive correlation was found between the amount of data produced (in GB) and the DNA concentration via the Spearman’s correlation. The data followed a positive monotonic relationship. However, when graphing the data, two possible lines of best fit were considered, one with a logarithmic trendline and the second with a linear trendline. In this instance, the Pearson’s correlation coefficient was >0.99 for the logarithmic line and >0.95 for the linear correlation. Although a positive correlation is seen in the amount of data produced, this was not used as a parameter for quantification because of the uncertainty between the two trendlines.

The N50 metric is related to the median and mean length of the sequences output from a run and measures the contiguity of an assembly, defined by the length of the shortest contig for which longer and equal length contigs cover at least 50% of the assembly [17]. The N50 values obtained from the sequencing runs for the five DNA concentrations were not significantly different as determined by a one-way analysis of variance (ANOVA). This suggested that the N50 values were not dependent on the amount of input DNA in our experiment [26,27].

**Figure 1:**
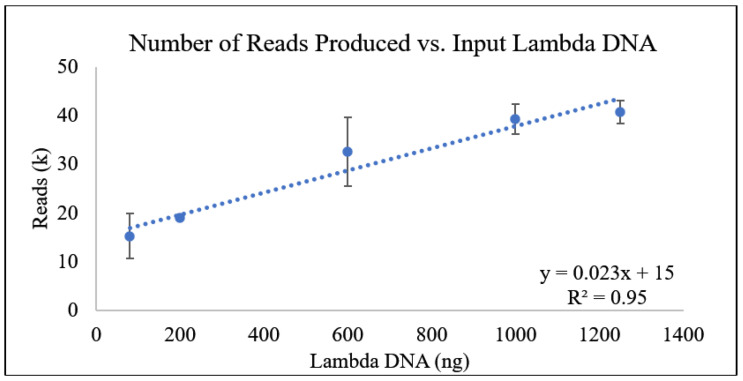
Amount of lambda DNA vs. the number of reads produced with relative error represented. Two lines of best fit are shown, a logarithmic and linear line.

The number of reads produced in each sample was then plotted against the amount of input DNA (Figure 1), and, like the amount of data produced, the Spearman’s correlation showed a monotonic relationship, and, in this case, a clear linear line of best fit.

Though the number of reads produced per run would not be considered a variable for quantification, particularly for a DNA sample generated from a mixed bacterial population, it did appear to demonstrate a positive linear relationship with the DNA quantities tested. The number of reads is dependent on, at minimum, two variables, the amount of input DNA and the length of the DNA fragments sequenced [28]. DNA is susceptible to physical and chemical breakage during preparation and storage, and in previous studies, it was found that this limits the length of the reads generated [29]. This suggests that replicates of the same DNA concentration could have variable distributions in their read lengths due to shearing during library preparation [30].

**Figure 2:**
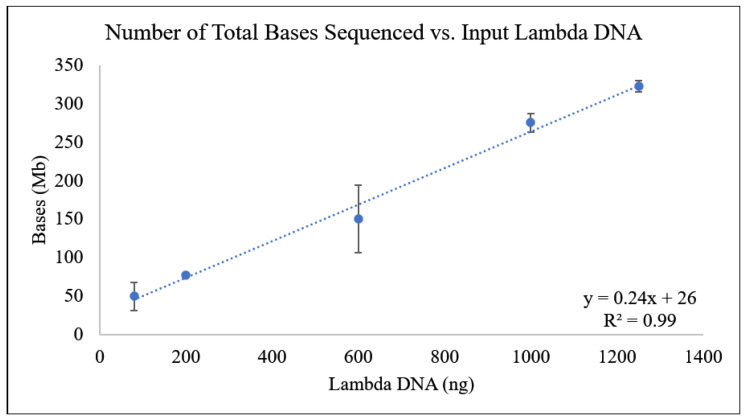
Amount of lambda DNA vs. the total amount of bases sequenced (Mb). A (linear) line of best fit was generated with this Equation: y = 0.24x + 26. The R^2^ value was 0.99, which suggested a strong correlation.

The amount of data produced showed a line of best fit, the N50 did not significantly change based on concentration, and the number of reads introduced length as another variable. The last determinant was the number of total bases sequenced in a run. Unlike a read, a nucleotide base is not variable by length and can be related directly to the lambda phage genome, as this experiment was conducted in the absence of any other DNA [31]. In this instance, a positive, distinctly linear correlation was derived (Figure 2), with the calculated slope of the line being 0.24 and an R^2^ value of 0.99. This result demonstrated that the number of bases sequenced could be used to quantify the input DNA sample through the generation of calibration curves. However, food samples rarely contain only one organism; therefore, further experiments were conducted to determine if quantification was still possible in the presence of background matrix DNA.

### 3.2. Spiked Bovine DNA General Run Outputs

Like the external standards methodological approach results presented in Table 1 and Figure 1, the default MinKNOW sequencing run parameters were used except for the minimum read length, which was set to 20 bp. In this set of experiments, a standard 1 µg input of *Bos taurus* DNA was spiked with different amounts of lambda phage DNA. The bovine and lambda DNA were combined and made up to volume with nuclease-free water prior to library preparation. The run time for each sample was set to 1 h. The nomenclature of the following runs represents the amount of spiked-lambda phage DNA. Table 2 shows the average values with standard deviation after outlier removal with the Dixon’s Q-test for each concentration of lambda DNA.

The number of sequences and bases mapped were determined from the lambda-spiked bovine DNA sequencing runs because they had exhibited a level of quantification in the external standards data set. When applying the same sequencing parameters to the lambda spiked bovine DNA raw data output, and comparing them to the other computed outputs, the number of sequences and bases sequenced continued to show quantitative potential following simple bioinformatics analyses (Table 2). Other raw outputs, such as the total data produced and N50, do not allow facile discrimination between the bovine and lambda DNA.

Upon first review of the output data, the total number of reads and the total number of bases sequenced do not show a clear trend. This was expected as each prepared library exceeded the recommended 1 µg DNA input and was therefore not limited by library depletion. In that case, it appeared that the pores of the sequencing chip were saturated at levels of DNA above 1 µg, and the number of total reads and totals bases sequenced quickly plateaued upon addition of lambda DNA to the background bovine DNA. Although a specific amount of each analyte was used in library preparation, we expected differential activity between the bovine and phage DNA during library preparation with the DNA end repair and ligation. This step would be the first phase of exclusion, as sequences that were not modified would not be sequenced [32]. Furthermore, it is not a guarantee that every prepared DNA strand would flow through a nanopore and be sequenced, this could be due to Brownian motion or other electrostatic/steric effects [32,33].

With these added variables, a post-run exclusion analysis was conducted with a relatively simple bioinformatics work-up, where the sequence reads were mapped to the complete *Enterobacteria* phage lambda reference genome (NCBI Accession #NC001416.1) using Minimap2 (version 2.24). For this mapping, the default parameters were used to generate a list of reads that successfully aligned to the reference genome. Using this list, the number of sequences and bases successfully mapped to the reference were calculated and used to normalize the general output data regarding the targeted sample (lambda). This normalization accounted for the data outputs produced within the sample groups and suggested which data sets were appropriate for quantification [34].

**Figure 3:**
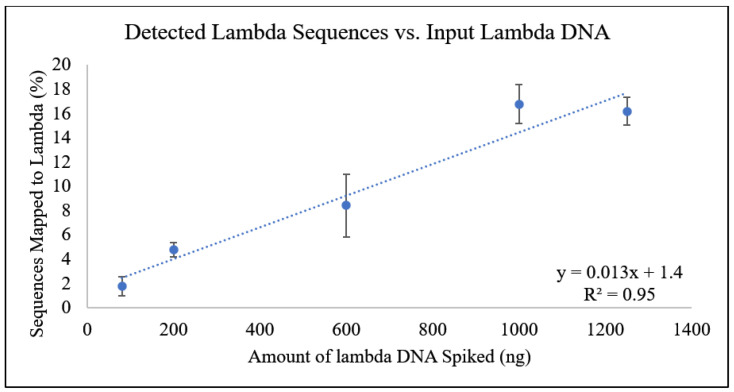
Amount of lambda DNA vs. the number of sequences mapped to lambda phage genome (%). A linear line of best fit was generated with this Equation: y = 0.013x + 1.4. The R^2^ value was 0.95.

Figure 3 shows a correlation between the amount of spiked lambda DNA and the percentage of all sequences that mapped to the lambda reference genome. This analysis revealed a clear linear relationship. This relationship was not seen between the raw data output of total reads and the lambda DNA concentration due to the presence of bovine DNA. All samples contained the 1 µg of bovine DNA plus increasing amounts of lambda DNA, most likely leading to a saturation of the sequencing pores. The results became dependent on the ratio of prepared lambda DNA/total DNA being sequenced in each run. However, the bioinformatics workup accounts for this ratio and allows for sequences to be identified as being derived from lambda.

**Figure 4:**
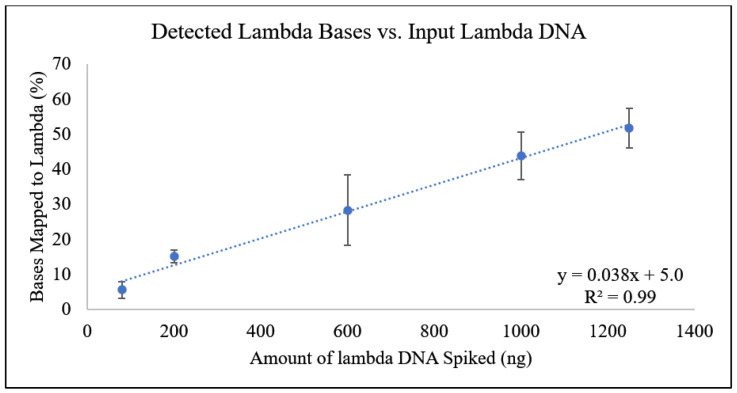
Amount of lambda DNA vs. the number of bases mapped to lambda phage genome (%). A linear line of best fit was generated with this Equation: y = 0.0381x + 5.06. The R^2^ value was 0.991.

The percentage of bases that mapped to the lambda reference genome in each tested concentration was also analyzed as a function of the amount of lambda DNA present in the sample (Figure 4). This comparison demonstrated a strong linear relationship (Figure 3). The sequenced DNA fragments vary by length and utilizing the number of bases rather than the number of reads removes this variable [29,30,31]. This analysis identified the number of bases belonging to lambda out of the total DNA sequenced and demonstrated that a level of quantification can be derived.

The generated calibration curves show that quantification is possible; however, the limit of detection (LOD) and limit of quantification (LOQ) were not determined during these experiments. Five different concentrations of lambda DNA were chosen to determine if quantification was possible using amounts of DNA that increased at irregular increments. The smallest non-zero amount of analyte investigated was 80 ng of lambda DNA. Further experimentation would be needed to test lower concentrations and potentially identify the LOD and LOQ.

**Figure 5:**
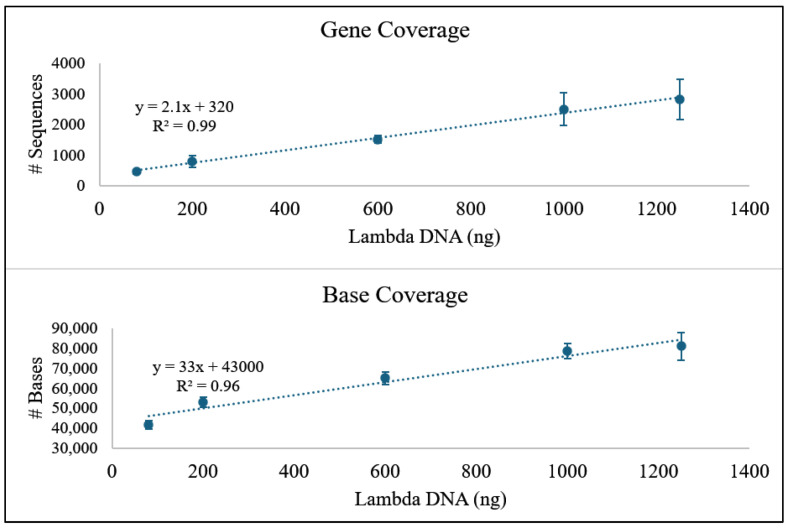
Amount of lambda DNA vs. coverage of the number (#) of sequences (**top**) and the number (#) of bases (**bottom**) to Lambdap22.

The possibility of detecting a particular organism by identifying a specific target gene was also examined by analyzing the data for sequences matching the lambdap22 sequence, which was 17,101 bp in length (approximately 35% of the lambda genome). Gene detection was examined by looking at the total number of sequences and the total number of bases that mapped to lambdap22. Two curves were generated to determine if there was a correlation with the amount of spiked DNA (Figure 5).

A positive correlation was observed between the amount of lambda DNA in the sample and the number of reads that mapped to lambdap22 in the presence of the bovine DNA. The same can be said for the number of bases that map to lambdap22 (Figure 5). This demonstrates a simple bioinformatic approach to detection and quantification. This same analysis can be used for other organisms, such as bacteria, if a reference genome is available, and a specific gene can be identified—for example, antibiotic resistance genes, virulence genes, serotype-specific genes, etc. [34]. While the present study was conducted as a proof-of-concept, it should be noted that the bioinformatic analysis conducted in this study (i.e., detection of the p22 sequence within the lambda genome) involved the detection of a large region in a relatively small genome. Detection and quantification of a foodborne pathogen would typically involve the detection of (a) much smaller gene(s) in a much larger genome. While several studies have been conducted to test the efficacy of ONT long-read sequencing for foodborne pathogen detection [10,13,14,15,16,17,18,19], these reports all rely upon culture enrichment, which precludes the quantification of pathogens from the food sample. Furthermore, many of these studies attempted partial or complete genome assembly to confirm pathogen presence [16,17,18,19]. Here, we conduct a basic set of experiments to simply test if ONT MinION sequencing yields a quantitative output that has the potential for application in pathogen quantitation.

The results for all experiments conducted support the quantitative capabilities of the Oxford Nanopore MinION in the presence and absence of background DNA. This study alludes to applications regarding food safety and can be extrapolated to applications such as pathogen identification and quantification.

## 4. Conclusions

Throughout this investigation, the quantitative capabilities of the Oxford Nanopore MinION were explored in both the presence and absence of background *Bos taurus* DNA using *Enterobacteria* phage lambda as a pathogenic bacterial surrogate. Current methods to detect and identify pathogens in swine, poultry, and cattle, such as enrichment culturing, can take days to obtain results, while rapid detection methods are often used in tandem to screen, quantify, and characterize samples for target pathogens, prolonging real-time comprehensive analysis [9,10]. Importantly, both traditional microbiological methods and rapid methods typically require selective culture enrichment in order to bring the levels of target pathogens to sufficient numbers for detection, thus eliminating the possibility of determining pathogen load in the food sample. Although sequencing technology is not often utilized in quantitative applications, the emergence and development of next-generation sequencing platforms that combine sequencing chemistry and bioinformatics technology provide the potential for quantitative applications [10,11,12,13,20]. The goal of this investigation was to explore quantitation via NGS through establishing a correlation between known amounts of analyte (lambda DNA) with data output, along with the identification of the analyte through targeted gene analysis. A level of quantification was identified that can be exploited for rapid foodborne pathogen detection. All sample conditions had a prep time of 1.2 h with pre-extracted DNA, a run time of 1 h, and post-sequencing analysis of 30 min, resulting in an approximately 2 h and 45 min detection, identification, and quantification pipeline.

Foodborne pathogen detection using long-read sequencing has multiple advantages over current methods. First, sequencing can be highly efficient when multiple samples are barcoded and run on one flow cell. Second, multiple pathogens can be bioinformatically targeted in a sample, reducing the labor needed for multiple culture-based tests. Third, one sequencing run can provide the data needed to determine serotype and antibiotic resistance gene carriage. Lastly, long reads facilitate genome assembly [35], while real-time analysis allows pathogen detection to be accomplished in real-time [36]. However, nanopore sequencing can be more error-prone, although significant progress has been made to improve raw read accuracy to >99.9% [37]. An accelerated basecaller, Dorado, that doubles basecalling speed has recently been released by ONT [38]. This will allow for the use of higher-accuracy basecalling models to find small genetic changes, such as single-nucleotide polymorphisms (SNPs), during real-time analysis of sequencing data [39]. Finally, the low concentration of pathogen DNA in relation to host DNA poses a challenge, and methods will need to be optimized to address this prior to sequencing during sample preparation or post-sequencing during data analysis. Future research endeavors will be informed by this proof of concept and will involve sequencing and quantification of pathogens in food samples, efforts to establish the limit of quantification and limit of detection, and investigation utilizing barcoding and DNA exclusion methods.

## Figures and Tables

**Table 1:** General MinION run outputs using varying amounts of lambda DNA.

Amount of DNA (ng)	Total Bases Sequenced (Mb)	Total # of Reads (k)	N50 (kb)	Data (Gb)
80	49.6 ± 31.9	15.2 ± 8.1	10.4 ± 2.8	0.9 ± 0.5
200	76.8 ± 1.4	18.9 ± 0.3	10.1 ± 1.6	2.2 ± 1.6
600	150.5 ± 74.7	32.5 ± 12.1	12.3 ± 3.6	3.6 ± 1.4
1000	275.6 ± 21.5	39.2 ± 5.3	12.9 ± 1.5	4.5 ± 0.4
1250	323.1 ± 12.0	40.8 ± 4.1	14.1 ± 0.9	5.2 ± 0.2

**Table 2:** Relevant MinION run outputs using varying amounts of lambda DNA in the presence of 1 µg of bovine DNA.

Amount Lambda DNA (ng)	Total # of Reads (k)	% Sequences Mapped to Lambda	Total Bases Sequenced (Mb)	% Bases Mapped to Lambda
80	87.2 ± 77.4	1.8 ± 1.3	276.7 ± 207.8	5.5 ± 4.0
200	139.8 ± 41.4	4.8 ± 1.0	394.4 ± 112.6	15.1 ± 3.2
600	149.1 ± 16.4	8.4 ± 4.5	396.3 ± 66.3	28.4 ± 17.3
1000	137. 4 ± 33.0	16.8 ± 2.8	403.0 ± 81.9	43.8 ± 11.6
1250	146.2 ± 44.6	16.2 ± 2.0	371.1 ± 213.3	51.8 ± 8.1

## Data Availability

The original contributions presented in the study are included in the article, further inquiries can be directed to the corresponding author.
